# Percutaneous Transluminal Renal Angioplasty for Fibromuscular Dysplasia and Prognostic Risk Factors: A Retrospective Chinese Cohort Study

**DOI:** 10.3390/jcm12010023

**Published:** 2022-12-20

**Authors:** Yi-Ting Lu, Ze-Ming Zhou, Di Zhang, Lin Sun, Xin-Chang Liu, Yan-Kun Yang, Xiong-Jing Jiang, Xian-Liang Zhou

**Affiliations:** 1 Department of Cardiology, Fuwai Hospital, National Center for Cardiovascular Diseases, Chinese Academy of Medical Sciences and Peking Union Medical College, Beijing 100037, China; 2 Department of Cardiology, Beijing Friendship Hospital, Capital Medical University, Beijing 100050, China

**Keywords:** fibromuscular dysplasia, percutaneous transluminal renal angioplasty, hypertension, stenosis, prognosis, risk factor

## Abstract

Fibromuscular dysplasia (FMD) is a non-atherosclerotic, non-inflammatory vascular disease involving small-to-medium-sized arteries. The characteristics of Chinese patients with FMD remain unclear. We retrospectively analyzed the data of patients with renal FMD who underwent percutaneous transluminal renal angioplasty (PTRA) for the first time at Fuwai Hospital between 2010 and 2021. The variables were selected through least absolute shrinkage and selection operator regression (LASSO), and logistic regression models were constructed to identify independent risk factors. A total of 116 patients (52 males, median age at diagnosis, 25.0 years) were enrolled. Elevated blood pressure was the leading complaint. After a median follow-up period of 18.0 months (interquartile range: 6.0–48.0 months), hypertension recurred in 34 patients and restenosis in nine patients, among whom four patients underwent secondary intervention and one patient underwent surgical revascularization. Bilateral renal artery involvement (odds ratio [OR]: 2.61, 95% confidence interval [CI]: 1.11–6.15; *p* = 0.028) and age at hypertension onset (OR: 0.93, 95% CI: 0.88–0.99; *p* = 0.018) were independent prognostic factors for adverse outcomes. The results indicate that patients with bilateral renal artery involvement and younger age at hypertension onset are more likely to have poorer clinical outcomes after PTRA, and should be more closely monitored.

## 1. Introduction

Fibromuscular dysplasia (FMD) is a segmental, non-atherosclerotic, non-inflammatory vascular disease that affects small-to-medium-sized arteries [1]. Histologically, FMD can be divided into three forms based on the affected vessel layer: (1) intimal fibrous circumferential intimal thickening presenting as solitary stenosis; (2) medial hyperplasia with the typical string-of-beads pattern; and (3) tubular-type adventitial fibroplasia [2]. Due to the inaccessibility of pathological tissues, angiography is routinely recommended for the diagnosis of FMD. Stenosis, occlusion, aneurysm, dissection, and arterial tortuosity are some of the morphological manifestations of FMD [1].

FMD can occur in people of all ages and can involve multiple vascular beds. However, it primarily occurs in middle-aged individuals, and the most frequently affected vessels are the renal and cerebrovascular arteries. Although the exact cause of FMD remains elusive, it is commonly recognized that the interaction of both genetic and environmental factors, including smoking, mechanical stimulation, and hormone factors, contribute to FMD pathophysiology and development [3,4,5]. The clinical presentation of FMD varies from asymptomatic to severe cardiovascular disease, such as headache, stroke, and myocardial infarction, amongst others [6,7,8]. The common symptom of FMD is hypertension [9,10], which is the second predominant cause of renovascular hypertension following atherosclerosis, accounting for approximately 10% of cases [11].

Presently, percutaneous transluminal renal angioplasty (PTRA) and surgery for revascularization are considered the most efficient treatment options based on the clinical symptoms of the lesions and the degree of stenosis. The small lesion size and possibility for re-intervention promote PTRA as the appropriate first-line treatment option for renal FMD.

Although several studies have investigated the short-to-long-term outcomes of PTRA in Western populations, related studies in Eastern populations are scarce. Previous studies have shown that Chinese patients differ from Western patients in terms of sex distribution and disease type, indicating that the Chinese FMD population might represent a separate entity [12,13]. Thus, we retrospectively analyzed the clinical characteristics and mid-term outcomes of patients with renal FMD at our institution and aimed to assess the effects of PTRA in Chinese patients with FMD.

## 2. Materials and Methods

### 2.1. Patients

The data of patients diagnosed with FMD at Fuwai Hospital between January 2010 and December 2021 were retrospectively reviewed. The inclusion criteria were (1) a diagnosis of renal artery FMD, and (2) renal artery intervention for the first time at our institution. FMD was diagnosed as nonatherosclerotic arterial encroachment or stenoses affecting the trunk or branches of medium size arteries based on catheter-based angiography, computed tomography angiography (CTA), magnetic resonance angiography (MRA), in the absence of aortic wall thickening, biochemical evidence of inflammation, and known syndromic arterial disease (neurofibromatosis type 1, Marfan syndrome) [1,14]. This study was approved by the ethics committee of Fuwai Hospital and was conducted in accordance with the Declaration of Helsinki. All the adult patients or legal guardian of the minors have signed the informed consent.

### 2.2. Treatment

The basic treatment of patients with FMD included antihypertensive medication to control blood pressure and revascularization, with PTRA being the first choice. Angiography was performed by experienced clinicians. PTRA was recommended if the diameter of the renal artery was reduced by more than 70% on angiography. PTRA was considered successful if residual stenosis was <30%; otherwise, patients were advised to undergo surgery or stent implantation. Renal function indices and blood pressure were measured using a catheter after PTRA.

### 2.3. Definition and Outcomes

**Figure 1:**
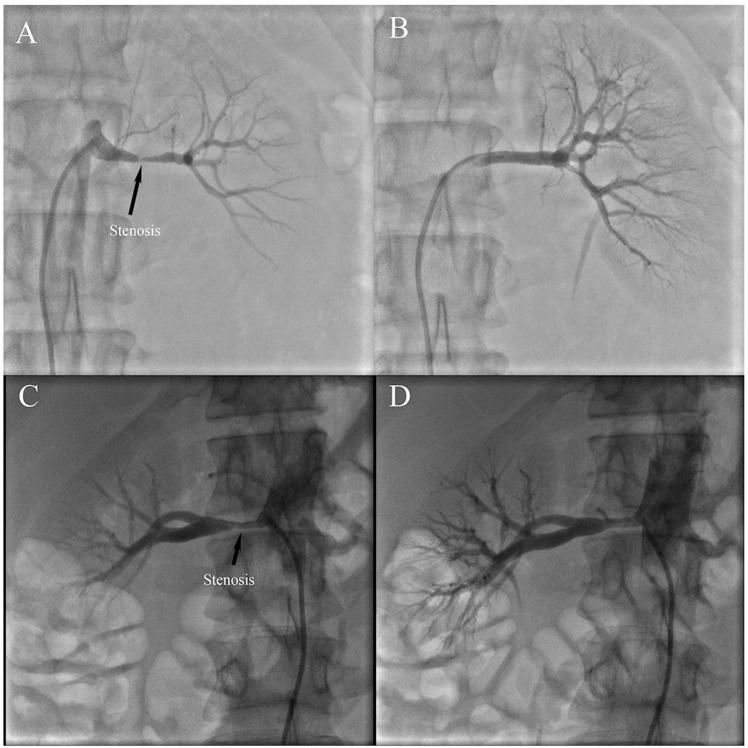
Focal fibromuscular dysplasia includes unifocal-type (**A**) and tubular-type (**B**). (**C**,**D**) representing vascular stenosis before and after percutaneous transluminal renal angioplasty, respectively. Arrows point to narrowed areas.

**Figure 2:**
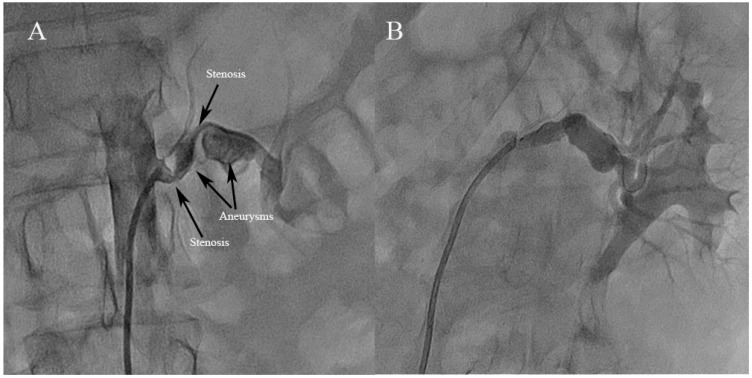
Examples of multifocal fibromuscular dysplasia simultaneously involving two stenoses on a given vascular segment and aneurysm (**A**,**B**) showing an improvement in the angiographic appearance as the result of successful percutaneous transluminal renal angioplasty.

**Figure 3:**
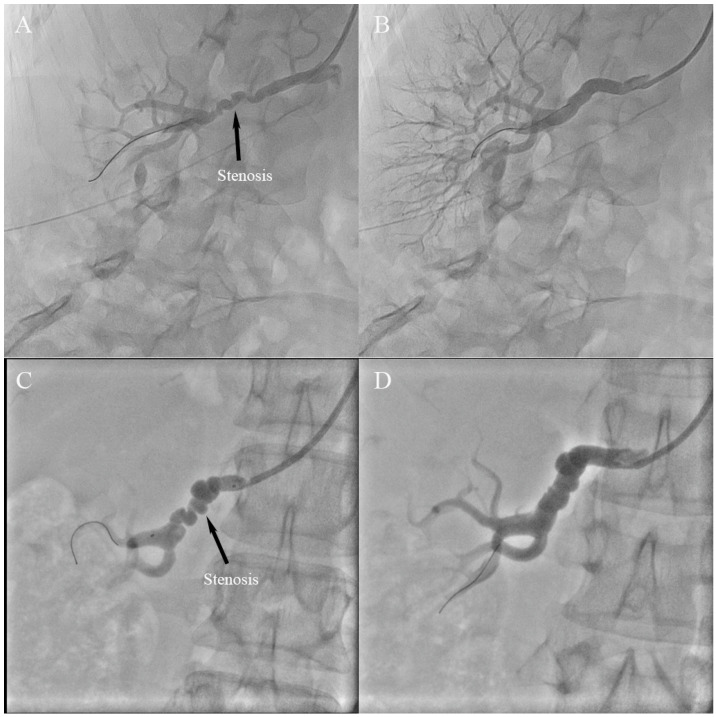
String-of-beads renal artery stenosis in a male patient with fibromuscular dysplasia. (**A**) the “string of beads” appearance of the renal artery and (**B**) post-successful percutaneous transluminal renal angioplasty of the lesion; (**C**) catheter-based angiography shows the recurrence of stenosis in the same patient after 8 years and (**D**) showing the result of successful reintervention.

Ostial lesions were defined as being within 0.5 cm of the main renal artery ostium, and proximal lesions were defined as being >0.5 and <1 cm from the renal artery ostium. Distal lesions were within 10 mm of the end of the main renal artery. Focal FMD was defined as isolated stenosis (Figure 1), comprising unifocal (<10 cm) and tubular-type (≥10 cm) [15]. Multifocal FMD was diagnosed in cases presenting with more than two stenoses on a given vascular segment (Figure 2) with or without the classic string-of-beads pattern (Figure 3) [16]. Aneurysm was defined as an affected segment that expanded >50% of the canal diameter (Figure 2). Hypertension was diagnosed in adult patients when blood pressure was ≥140/90 mmHg, and in children when greater than age-, sex-, and height-specific 95th percentiles [17].

Outcomes, including all-cause death, hypertension recurrence, restenosis/revascularization, and stroke were investigated during follow-up. Recurrent hypertension was defined as recurrence of a diastolic blood pressure of ≥90 mmHg, a systolic blood pressure of ≥140 mmHg based on daily regular measurements, or a ≥20% increase in post-procedural diastolic blood pressure that was not treatable by repeat angioplasty [18]. Restenosis was defined by recurrent stenosis of ≥50% after stent placement or successful PTRA, as demonstrated by CTA, MRA, or angiography [18].

### 2.4. Data Collection

The clinical information of the enrolled patients was collected, including demographics, symptoms, comorbidities, family history, and laboratory data. All data related to PTRA were collected through the electronic medical records system.

### 2.5. Follow-Up

All patients underwent education and standard training of automated upper arm blood pressure measurement devices before discharge. The baseline blood pressure of the patient was defined as average blood pressure standardly measured at home within one week after discharge by means of the automated upper arm devices, and the same device was used for blood pressure monitoring during follow-up. Home blood pressure level is the average of all blood pressure readings performed with an automatic upper arm device, for 6–7 consecutive days, with readings in the morning and the evening, taken in a quiet room after 5 min of rest, with the patient seated with their back and arm supported. Two measurements should be taken at each measurement session, performed 1–2 min apart. All the devices used had been validated according to agreed-upon criteria, which meet the validation protocols of the American Association for the Advancement of Medical Instrumentation and the British Hypertension Society [19,20]. As for the patients enrolled in the early study, the baseline data of home blood pressure was lacking. Therefore, in these patients, the past blood pressure over the years and hypertension-related clinical symptoms were collected. All the patients underwent ultrasound at 1, 3, 6, and 12 months, and repeat multi-detector CT as necessary. Patients were followed up by telephone or by a visit to the outpatient department.

### 2.6. Statistical Analysis

SPSS (version 26.0) and R (version 4.2.0) software were used for the statistical analyses. Data are presented as the mean ± standard deviation or as the median and interquartile range (IQR). Categorical data are presented as frequency (percentage). The Wilcoxon rank-sum test was used to compare the clinical data of patients before and after PTRA. Risk factor estimation of the outcomes was performed using a combination of least absolute shrinkage and selection operator (LASSO) and univariate/multivariate logistic regression analyses presented as measures of association. A *p* value of <0.05 was considered statistically significant.

## 3. Results

### 3.1. Patient Characteristics

A total of 136 patients were diagnosed with renal artery FMD between January 2010 and December 2021 at Fuwai Hospital. Seven patients who did not undergo PTRA, two patients with concomitant atherosclerotic renal artery stenosis (both age >50 years with cardiovascular risk factors including hyperlipemia and diabetes), five patients with concurrent severe extrarenal FMD, three patients who underwent PTRA for the first time at other hospitals, and three patients in whom PTRA failed due to failure of the guidewire to pass through the stenosis were excluded. Finally, the data of 116 eligible patients diagnosed with renal artery FMD who underwent renal artery intervention for the first time were included and analyzed. The patient characteristics are presented in Table 1. The median diagnose age of the patients was 25.0 years (IQR:19.0–31.0 years), and 89 patients were ranged in age from 18 years to 40 years.

### 3.2. Angiographic Findings and Treatment

A total of 119 patients underwent angiography, and the technical success rate of PTRA was 93.3% (111 of 119). The eight patients with technical failure included three patients in whom the guidewire failed to pass through the stenosis and five patients with residual stenoses of >30% and <50% after PTRA. Excluding the three patients in whom the guidewire failed to pass through the stenosis, the detailed information about 116 cases of renal artery involvement is displayed in Table 2. Six patients also underwent stent implantation because of technical failure. Sixteen patients underwent bilateral renal artery PTRA, five of whom presented with unilateral string-of-beads stenosis, four of whom had focal lesions, and seven of whom had bilateral string-of-beads stenosis. Table 3 shows that the post-intervention blood pressure values and mean creatinine, blood urea nitrogen, and uric acid concentrations were significantly lower than the pre-intervention values.

Previous studies performed in the Western population have shown significant differences in terms of age at hypertension diagnosis and at the first intervention compared to the present study in Chinese patients. Thus, based on the present population, we divided the patients into two groups based on their age at first FMD diagnosis (≥30 years and <30 years). In this subgroup analysis, the duration of hypertension in patients aged ≥30 years was longer than the duration of hypertension in the group aged <30 years (*p* = 0.007). Moreover, the degree of stenosis was smaller in ≥30 (*p* = 0.047) (Table 2). The duration of hypertension was relative to the time of diagnosis (Spearman Rho = 0.31, *p* = 0.001) in all patients.

### 3.3. Follow-Up and Outcomes

**Figure 4:**
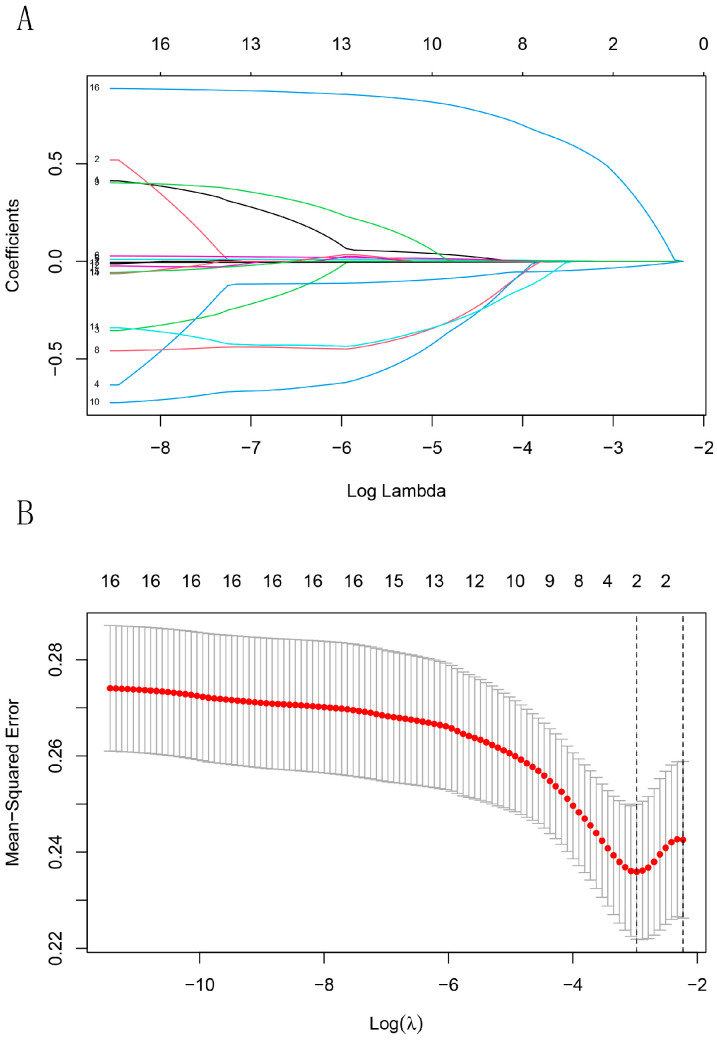
Least absolute shrinkage and selection operator (LASSO) analysis. (**A**) LASSO regression was used to screen the prognostic factors. Coefficients of the determined characteristics are exhibited via lambda parameters. (**B**) Cross-validation indicated that two factors (bilateral renal artery and age at hypertension onset) can be used in logistic regression.

The median follow-up period was 18.0 months (IQR: 6.0–48.0 months). In diagnosing hypertension recurrence, we also referred to the blood pressure level at discharge to avoid overdiagnosis, especially in the early time enrolled patients. Finally, hypertension recurrence occurred in 34 patients, two of whom suffered from stroke. Restenosis occurred in nine patients, of whom four underwent secondary intervention and one underwent surgical revascularization. The potential risk factors for events were selected using the LASSO method (Figure 4) and predicted using the univariate and multivariate logistic regression models (Table 4). Bilateral renal artery involvement (OR: 2.61, 95% CI: 1.11–6.15; *p* = 0.028) and age at hypertension onset (OR: 0.93, 95% CI: 0.88–0.99; *p* = 0.018) were significant independent prognostic factors for adverse outcomes in both models. Considering that Chinese FMD patients may be an independent group, which is different from the Western population, we divided the patients into two groups based on the median age at hypertension onset of 21.5 years. The subgroup analysis showed that age at hypertension onset of <21.5 years (OR: 3.14, 95% CI: 1.42–6.94; *p* = 0.005) was a significant independent risk factor for adverse outcomes, and the baseline characteristics of ≥21.5 years and <21.5 years groups was not significantly different. The results also show that the lesion in the subgroup with bilateral renal artery involvement was longer (29.56 ± 20.76 vs. 17.10 ± 10.48 mm; *p* = 0.001) and multifocal-type disease was more common (53.1% vs. 16.7%; *p* < 0.001) than in the unilateral renal artery subgroup.

## 4. Discussion

The present study retrospectively analyzed the clinical data of a large sample of East Asian individuals with FMD with a mid-to-long-term follow-up period. Using these data, we evaluated the PTRA outcomes to identify the risk factors that might affect prognosis.

### 4.1. Comparison of Eastern and Western Patients

Since FMD was first described in 1964 by Palubinski and Ripley in a woman with evidence of internal carotid artery fibromuscular hyperplasia [21], several studies have indicated that females account for the majority of affected patients. However, differences exist between Western and Eastern patients. With the widespread utilization of CTA/MRA and angiography for the diagnosis of FMD in clinical practice, the prevalence of FMD has increased. This is contrary to the previous impression that FMD is a rare disease [22]. Based on kidney donor data, the prevalence of FMD in the potential kidney donor population is approximately 3–4% [23]. However, currently, epidemiological studies in East Asian populations are lacking. In the Cardiovascular Outcomes in Renal Atherosclerotic Lesions Trial (CORAL), Hendricks et al. identified that 5.8% of 997 patients with hypertension had FMD on review of angiographic imaging [24]. FMD can occur at any age, but it predominantly affects middle-aged women [23]. A multicenter, large-scale study conducted by the European/International FMD registry and Initiative (FEIRI) revealed that the average age at FMD diagnosis is 46 ± 16 years, with a female to male ratio of 8:2 [25]. Another United States registry report found that the mean age at FMD diagnosis was 51.9 ± 13.4 years, with women accounting for 91% of cases [6]. However, in the current study, the mean age at FMD diagnosis was 25.12 ± 7.97 years. Of the affected patients, 44.8% were male, which is similar to previous reports by Yang et al. (mean age: 28 years, age range: 13–64 years, 43.8% male) [12], Chen et al. (26.7 ± 8.2 years, 50.5% male) [13], and Li at al. (18.9 ± 6.0 years, 54.1% male) [26]. The younger age at diagnosis and no obvious sex predilection indicates that the Chinese FMD population might be distinct from the Western population. A history of smoking/current smoking was noted in 16.4% of patients in our cohort, which is substantially lower than the rate of 62% in the French-Belgian Assessment of Renal and Cervical Artery Dysplasia (ARCADIA) international registry [27] and 37.2% in the United States registry [6], probably owing to the overall younger age of our cohort.

In both Western and Eastern patients, hypertension is one of the most frequent presenting symptoms associated with renal FMD [28]. Based on data from the FEIRI, among 1020 patients with FMD, 72% of patients presented with hypertension [25]. Similarly, 322 of 447 patients (72%) had hypertension in the United States registry study [6], and 363 of 469 patients (77.4%) presented with hypertension in the ARCADIA study [27]. In the present study, all patients had hypertension, and six patients suffered from stroke due to poor blood pressure control. The reason the percentage of patients with hypertension was higher than in previous reports is ascribed to the inclusion criteria of renal FMD evidenced by angiography, CTA or MRA. Moreover, in the pediatric FMD population, the prevalence of renovascular-related hypertension was 100% [29]. Most patients who received the initial diagnosis were transferred from other institutions for further diagnosis and treatment. The first consideration of hypertension in adults is artery atherosclerosis, rather than FMD. Therefore, older patients suffer from longstanding hypertension and misdiagnosis until the final diagnosis. The results of this study support this point. Therefore, it is proposed that routine screening for renal FMD should be performed in young patients with hypertension [30]. In addition, certain symptoms, such as dizziness and headache, are also common features of renal FMD [31], which were observed in 38.8% and 31.9% of patients in the present study. As patients with headache tend to have extracranial carotid or vertebral artery FMD, although we ruled out these conditions, these patients should undergo routine carotid ultrasound to monitor FMD progression [32]. However, it should be noted that carotid FMD lesions are not easily accessible to ultrasound because they are too distal.

### 4.2. Technical Advantages of PTRA

Although pathology is the gold-standard diagnostic method, catheter-based angiography is of great value as it can provide more details about FMD. According to angiographic images, FMD can be classified into multifocal and unifocal forms, of which the former is the most common type in Western FMD populations [33]. Of note, in this study, unifocal FMD accounted for 82.8% of cases, which is contrary to previous reports. Aneurysm was present in 20 of 116 patients (17.2%) in our cohort, which is close to the proportion of 21.7% reported by Kadian-Dodov et al. [34]. The reason the prevalence of aneurysm in our FMD cohort was slightly lower might be because systematic CTA screening was not performed in every patient; thus, some vascular lesions may have remained undetected. Moreover, the middle renal artery is the most involved site of FMD, followed by the proximal and distal renal arteries [35]. All of these observations describe the characteristics of renal FMD, and are useful for identifying FMD and distinguishing it from other stenotic diseases.

PTRA is considered the first-line treatment for renovascular hypertension caused by FMD. In the present study, the technical success rate of PTRA was 93.3%, and the effects on blood pressure and renal function, including creatinine, blood urea nitrogen, and uric acid concentrations, decreased significantly after PTRA. Of note, the decrease in plasma creatinine levels after PTRA was slight, which may be associated with the occurrence of early kidney injury in patients with chronic severe hypertension and the use of contrast media during PTRA procedure. With the increase in research on FMD, the number of studies on the efficacy of PTRA in patients with renal FMD is gradually increasing. A meta-analysis of 47 angiography studies involving 1616 patients with FMD revealed that the hypertension cure rate after revascularization was 40%–52% [36]. Moreover, an analysis of 22 patients with renal FMD after PTRA showed that the rates of freedom from recurrent/worsening hypertension were 89.4%, 89.4%, 81.3%, and 71.1% for each additional year [37]. Alhadad et al. reported that the primary patency rate with PTRA revascularization was 73% at 12 months. Both blood pressure (from 174 ± 33 mmHg/100 ± 13 mmHg to 140 ± 25 mmHg/83 ± 12 mmHg; *p* < 0.0001) and the number of antihypertensive drugs (from 2.3 ± 1.2 to 1.6 ± 1.5; *p* = 0.0011) decreased after undergoing PTRA during the 7-year follow-up period [38]. The long-term outcomes of PTRA efficacy for FMD demonstrated that the restenosis rate was 28% at 5 years and 50% at 9 years [39]. In this relatively large study of 116 patients, the technical success rate was over 90%, and 63% of patients have controlled blood pressure during 18 months of follow-up, further supporting the conclusion that PTRA is a safe and useful option for treating renovascular hypertension in patients with FMD.

### 4.3. PTRA Prognosis

All patients face the risk of restenosis, which requires re-intervention after PTRA. The statistical analysis indicated that bilateral renal artery involvement and younger patients had a higher risk of restenosis. We also found that a younger age at hypertension onset increased the likelihood of mid-term adverse outcomes. A meta-analysis conducted in 2010 revealed that the mean age was younger among cured patients [36], which is contrary to the results of our study. There are several possible reasons for this. First, only a small number of patients (89 patients) from two trials were included in the subgroup analysis of the meta-analysis focusing on the relationship between mean age and the effect of PTRA, which might not be representative. Second, the patients were much younger in this study than in the meta-analysis, and only patients with renal FMD who underwent PTRA were included in our study. The majority of patients were referred to our hospital to identify secondary hypertension, and we only recruited three patients aged >40 years to avoid the influence of other potential factors, such as atherosclerosis. Young patients were selected as a priority. Finally, as mentioned above, the Chinese FMD population is different from the Western population in terms of sex and FMD type. A previous subgroup analysis of younger patients (<21.5 years) indicated that a young age was an independent risk factor for a high restenosis risk. We suggest that the poorer response to PTRA in younger patients may be due to the smaller vessel diameter and immature vascular development factors [40]. Thus, timely diagnosis of renal FMD combined with hypertension and the adoption of interventional measures are of great importance, and more attention should be paid to patients with early-onset FMD.

We found that bilateral renal artery involvement was a risk factor for adverse outcomes after PTRA. In this study, unilateral stenosis (72.4%) occurred more frequently than bilateral stenosis (27.6%), which is similar to a previous report by Alhadad et al. [38]. Studies that have discussed the significance of bilateral lesions in terms of the medium-term outcomes of renal FMD are scarce. As reported by Novick and colleagues, among seven patients with renovascular hypertension and bilateral renal artery disease, only two patients were cured of hypertension after surgery, one patient improved, and four patients demonstrated failure [41]. In terms of renovascular hypertension caused by Takayasu arteritis, it is evident that bilateral lesions are significant adverse prognostic factors [42]. In consideration of our results, the efficacy of PTRA decreased in patients with bilateral renal FMD, which may have been due to renal artery lesion length and multifocal-type, which is associated with longstanding kidney damage [43]. Meanwhile, compared with unilateral lesion, bilateral lesions lack functional compensation of the contralateral kidney. Thus, bilateral lesions faced a higher risk of endpoint events such as hypertension recurrence.

This study has some limitations that should be noted. First, this is a single-center retrospective study with inescapable referral bias, which stemmed from the transfer of patients from subordinate hospitals. Second, we only examined the mid-term follow-up records of these patients; thus, further monitoring of long-term follow-up records should be performed in the future. Third, the data on age at hypertension onset and hypertension duration were collected from the patient’s chief complaint records; thus, the time of severe renal artery stenosis may not be accurate. Fourth, except for the nine restenosis and two stroke patients, other patients’ blood pressure levels during follow-up were obtained from home blood pressure monitoring. Although we conducted patient education and training before discharge, home blood pressure monitoring compliance was not optimal in all patients. Finally, baseline home blood pressure was lacking for the patients enrolled in the early period. We nevertheless collected their office blood pressure, and medical records and referred the pressure to the office blood pressure at discharge when diagnosing the outcome.

## 5. Conclusions

In summary, FMD is a heterogeneous, systemic, non-inflammatory, non-atherosclerotic disease of the arterial wall, which often involves the renal arteries. We report in detail the characteristics of 116 Chinese patients with renal FMD and show that PTRA is an effective treatment strategy. Younger age at hypertension onset and bilateral renal artery involvement might be associated with poorer clinical outcomes. However, this remains to be verified in large-scale and prospective studies.

## Figures and Tables

**Table 1:** Patients’ demographics, symptoms, comorbidities, and family history.

Variables	Total (N = 116)
Males, n (%)	52 (44.8)
Age at FMD diagnose, median (IQR), years	25.00 (19.00, 31.00)
Age at HT onset, median (IQR), years	21.50 (17.00, 27.00)
BMI, median (IQR), kg/m^2^	20.70 (19.10, 24.76)
Signs, n (%)	
Headache	37 (31.9)
Dizziness	45 (38.8)
Hypertension	116 (100.0)
Hyperlipidemia	11 (9.5)
Stroke	7 (6.0)
Family history of HT, n (%)	47 (40.5)
History of smoking/current smoking, n (%)	19 (16.4)
History of drinking/current drinking, n (%)	9 (7.8)

FMD, fibromuscular dysplasia; HT, hypertension; BMI, body mass index.

**Table 2:** Angiographic results of patients with FMD.

Characteristics	Total (N = 116)	<30 Years Group (N = 78)	≥30 Years Group (N = 38)	*p* Value
Age of hypertension onset, median (IQR), years	21.50 (17.00, 31.00)	18.75 (14.50, 22.00)	29.85 (25.75, 33.25)	<0.001
Type, n (%)				0.61
Unifocal	85 (73.3)	56 (73.7)	29 (73.2)	
Multifocal	31 (26.7)	22 (28.2)	9 (23.7)	
Aneurysm, n (%)	20 (17.2)	13 (16.7)	7 (18.4)	0.81
Renal artery branch, n (%)	35 (30.2)	24 (30.8)	11 (28.9)	0.84
Ostial or proximal lesion, n (%)	37 (31.9)	24 (30.8)	13 (34.2)	0.71
Distal lesion, n (%)	25 (21.6)	15 (19.2)	20 (26.3)	0.38
Degree of narrowing, median (IQR), %	90 (80, 95)	90 (80, 95)	90 (80, 90)	0.047
Lesion length, mean ± SD, mm	20.52 ± 15.06	19.62 ± 14.61	22.42 ± 15.99	0.35
PTRA + stent, n (%)	6 (5.2)	3 (3.8)	3 (7.9)	0.39
Duration of hypertension, mean ± SD, years	3.10 ± 4.32	2.16 ± 2.81	5.04 ± 5.99	0.007
Bilateral Renal Involvement, n (%)	32 (27.6)	21 (26.9)	11 (28.9)	0.82

The *p* value is the comparison of patients’ FMD diagnosis age of ≥30 years and <30 years. PTRA, percutaneous transluminal renal angioplasty.

**Table 3:** Blood pressure condition, renal function index of FMD patients before and after intervention.

	Pre-Intervention	Post-Intervention	*p* Value
SBP, median (IQR), mmHg	159.50 (146.50, 170.75)	132.87 (120.75, 140.00)	<0.001
DBP, median (IQR), mmHg	97.00 (84.00, 106.00)	80.00 (75.00, 89.00)	<0.001
CREA, median (IQR), umol/L	67.96 (61.13, 83.10)	66.55 (58.53, 83.33)	0.005
BUN, median (IQR), mmol/L	4.87 (4.10, 5.71)	3.43 (2.90,4.26)	<0.001
URIC, median (IQR), umol/L	304.80 (254.94, 396.01)	269.21 (198.15, 343.74)	<0.001

SBP, systolic blood pressure; DBP, diastolic blood pressure; CREA, creatinine; BUN, blood urea nitrogen; URIC, blood uric acid. Differences were considered statistically significant if *p* < 0.05.

**Table 4:** Prognostic factors for adverse outcome under univariate and multivariate logistic analyses.

	Univariate Logistic Analysis			Multivariate Logistic Analysis		
	OR	95% CI	*p* Value	OR	95% CI	*p* Value
Bilateral renal artery	2.528	1.098–5.823	0.029	2.610	1.107–6.153	0.028
Age at HT onset	0.935	0.884–0.989	0.019	0.932	0.880–0.988	0.018

## Data Availability

Not applicable.
